# Supplementary material for: Are carbon emissions trading and green financial instruments synergistic? -Comprehensive quantitative research based on content analysis
Source: PLoS One. 2024 Mar 7;19(3):e0298601. doi: 10.1371/journal.pone.0298601 (PMC10919601; doi:10.1371/journal.pone.0298601)
Supplement: S1 Data — (ZIP) [file pone.0298601.s001.zip › Data/地级市Z_F工作报告.zip!/╔┬╬≈/░▓┐╡╩╨/└┤╫╘╣1⁄2╓┌║┼í╛┬φ┐╦╩2╛▌═°í┐_2017.docx]

各位代表：

  现在，我代表市人民政府向大会报告工作，请予审议，并请市政协委员和其他列席人员提出意见。

  一、2016年和本届政府工作回顾

  2016年，我们以新发展理念为引领，聚焦“五个扎实”，矢志追赶超越，加快推进绿色循环发展，实现了“十三五”稳健开局。全市生产总值852亿元，增长11.3%；财政总收入78.3亿元，增长8%，一般预算收入30.1亿元，同口径增长12%；城乡居民收入达到25962元和8590元，分别增长8.2%和8.6%，单位生产总值能耗下降4%。

  经济运行稳中向好。生产总值、第二产业增加值、规模工业增加值增速和非公经济占GDP比重等主要指标居全省前列。完成全社会固定资产投资926.4亿元，增长24.2%，20个重点产业项目建成投产，安康机场、平镇和安岚高速、阳安铁路复线等重大基础设施项目开工，酒泉--湖南±800千伏特高压输电工程安康段建成。富硒特色产业增长24%,富硒茶科研成果在人民大会堂成功发布，魔芋产业发展“安康模式”被全国推广，农业部批准在我市建设富硒产品开发与质量控制重点实验室。规模工业总产值突破千亿，增加值增长16%，居全省第一；以奥邦锻造、南水汽配等为重点的智能制造取得突破。现代服务业加快发展，新增限额以上商贸企业150户，社会消费品零售总额增长14%。旅游接待人数和综合收入分别增长15%和18%；紫阳、平利跻身国家电子商务示范县，石泉、岚皋、宁陕创建国家全域旅游示范区，瀛湖生态旅游区成为国家服务业标准化试点景区。

  发展活力持续增强。创新驱动战略和“双创”行动计划顺利实施，科技创新引导基金和产业发展基金助推创新发展。建成秦巴众创空间等7个国家级“双创”平台和富硒产品研发中心等6个重点研发机构，培育高新技术、民营科技企业140家。开放发展“十大行动”成效明显，举办经贸、外事、文体等活动近百场次，在天津、武汉、沈阳、成都等地组织推介招商活动20多场次，驻外使节来安考察、驻华使节文化（安康）行活动产生良好影响，中日地方交流研讨会首次在非省会城市举办，首届中国创新挑战赛（安康赛区）、国际富硒魔芋贸洽会、第十六届汉江龙舟节等活动取得圆满成功。招商引资项目276个，到位资金458.6亿元。深化财税金融改革创新，安康被确定为国家四个政策性金融扶贫实验示范区之一和省级金融精准扶贫示范区、绿色金融示范市，平利县“两权”抵押贷款试点获得全国人大法律授权；高新金融服务聚集区启动建设，13户企业在股权交易市场挂牌，金融机构存贷款余额分别增长13.5%和14.6%。深入推进“放管服”改革，市场活力进一步释放，新增注册企业4878户，增长40%。

  新型城镇化步伐加快。统筹中心城市、县城、重点镇、新型社区和美丽乡村建设，城镇化率提高到45.6%。中心城市建设完成投资54亿元，金州路、兴安路（一期）和316国道江北段提升改造、东坝汉江大桥、环江50里慢行系统（一期）、8个公交港湾、500个停车泊位等市政工程建成投用。新投放公交车50辆，开通城区至县河等公交线路。腾退机关事业单位房地产近4万平方米建设公共服务设施。职业技术学院整体北迁，城区中小学校新增校舍37.5万平方米、运动场6.9万平方米，新增学位1.5万个。市体育综合训练馆建成投用。成功创建国家“双拥”模范城。县城和县域副中心镇、文化旅游名镇、新型社区建设加快，恒口镇级小城市综合改革试验区挂牌运行，平利美丽乡村标准化建设走在全省前列。开工建设保障性住房20919套，分配入住22788套；全面放开城镇户籍，农村居民进城入镇8.4万人。

  生态建设成效显著。深入推进国家主体功能区建设试点示范，坚持山水林田湖一体化治理，造林绿化68万亩，退耕还林18万亩，治理水土流失845平方公里，森林覆盖率65%。汉江水质保护十大专项行动和“治污防霾•保卫蓝天”成效显著，建成南水北调环境应急处置中心、陕南环境应急物资储备库和6个水质自动监测站、10县区环境空气质量监测站，新建和改造垃圾污水处理设施47个。“气化安康”稳步推进，建成加气站8座，新增用户3.2万户。强力推进节能减排，建成94个重点减排项目、18个污染治理和节能技改项目。汉江水质稳定保持国家Ⅱ类标准，环境空气质量综合指数居全省第一，空气优良天数达301天，比上年增加14天；被确定为全国第一批医养结合试点市和省级生态健康养老示范市。

  民生和社会事业长足发展。完成民生支出206.3亿元，增长10.8%。民生实事总体完成，200个贫困村12.2万贫困人口脱贫目标全部实现。新增城镇就业2万人，劳动力转移就业64万人。完成教育投资6.5亿元，学前三年教育普及率96.3%，高中阶段入学率96.5%，高考一、二本上线率全省第二。建成市人民医院门诊综合楼和市精神病专科医院，选派2181名教育、医务工作者到国内外培训深造。安康博物馆获第十三届全国博物馆十大陈列精品奖，安康第二博物馆、汉江龙舟文化园等建成投用。建成镇、村（社区）体育惠民工程450个，全民健身及场地建设走在全省前列，成功承办陕西省首届全民健身大会。推进市级老年公寓和15个社区日间照料中心、100个农村互助幸福院建设。城镇企业职工基本养老保险、失独家庭扶助关怀和城乡居民养老、医疗保险制度实现全覆盖。强化社会治安防控，完善联控平台和巡防体系。安全生产、防灾减灾、食品药品监管和依法治访工作扎实有效，社会大局和谐稳定。

  过去一年，我们深入开展“两学一做”学习教育，以在党爱党的坚定和追赶超越的行动，检验“学”的效果，树立“做”的标杆，打造“四讲四有”过硬公务员队伍。自觉接受人大、政协监督，主动报告、通报重大事项。坚定不移地推进政府廉洁建设，行政监察和审计监督力度加大，政风行风持续改进。扎实推进依法行政，深入实施“放管服”改革，加快简政放权和职能转变，公开承诺的1012项任务基本完成。政府其他各方面工作也取得了新的成绩。

  各位代表，2016年工作的全面完成，为本届政府任期划上了圆满句号。过去六年，在宏观环境复杂、经济持续下行情况下，我们依靠全市人民的智慧和力量，埋头苦干，砥砺前行，为全面建成小康社会打下了坚实基础。

  一是实现了由滞后发展向追赶超越的重大转变。全市经济年均增长13%，高于全省3个百分点。经济总量是2010年的2.6倍。全社会固定资产投资3457亿元，年均增长25%。非公有制经济占GDP比重55.5%，金融机构存贷比54%，经济运行中有潜力、有活力的积极因素增多。全面小康指数提高到77.54%，年均增长3.5个百分点，主要经济指标增速保持全省第一方阵，9县区12次获得全省县域经济“争先进位”奖。

  二是取得了一批增实力蓄势能的重要成果。产业支撑能力增强，工业保持强劲增长，建筑业产值突破百亿，现代农业园区发展到427个，生态友好型产业占比达65%，产业结构从2010年的20.5:40:39.5调整到11.8:54.8:33.4。安康高新区升级为国家高新区，旬阳工业集中区被批准为省级高新技术产业开发区，汉滨、汉阴、旬阳、平利工业集中区产值均突破百亿。富硒产业连年保持高速增长，从基地、产品到质量标准、品牌建设都取得重大进展。旅游、文化、体育、健康、养老“五大幸福产业”蓬勃发展，多点支撑、多元带动的产业格局正在形成。

  三是突破了基础设施滞后的瓶颈制约。建成十天、安平高速公路、西康铁路复线、平镇二级公路、安康中心港和5座汉江大桥，新建和提等改造县镇村公路近万公里，西成客专安康段、大岭铺至安康铁路直通线工程即将竣工，长期的交通“短板”正在转化为助推开放发展的区位优势。汉江综合整治、中小河流和病险库塘治理等项目加快推进，东坝防洪工程建成投用，长期危害安康的“水患”正在变为“水利”。农业生产、电力、电信、市政等领域的一批重大项目建成，绿色循环发展的基础保障能力明显提升。新型城镇化进程加快，四级城镇规划体系基本建立，中心城市、城镇建成区分别达39.5和91.9平方公里。中心城市创建为国家卫生城市，省级园林县城、卫生县城实现全覆盖。创建2个国家级生态示范区和6个省级生态县，生态文明建设走在全省前列。

  四是激发了改革创新与开放发展的生机活力。强力推进简政放权和职能转变，取消和下放行政审批216项，建成市县镇三级政务中心，紫阳“一站式”服务模式在全省推广。深化国有企业改革，完善法人治理结构，建立市属国有企业负责人薪酬管理、业绩考核、股权代表管理等制度。科技支撑能力增强，申请专利2600件，技术合同成交额1.6亿元。发展地方性融资机构40家，发行企业债券19亿元。政府机构改革、农村综合改革以及教育、卫生、公安、国土、交通等重点领域改革取得实效，一批改革试点和创新实践获得中省肯定。全方位推进对外开放，安康美食和特色产品走向全国各地，大唐电力、沈阳机床、上海复星、中建、陕建等30多家500强企业落户安康。引进域外资金1900亿元，年均增长30%。

  五是增强了保障和改善民生的工作实效。坚持把民生幸福作为施政追求，每年办成一批顺民意、惠民生的实事。强力推进扶贫开发和移民搬迁，减少贫困人口54.5万人，移民搬迁71万人。城镇累计新增就业10.7万人。创建国家义务教育均衡发展县7个、省“双高双普”县8个、教育强县4个。完成卫生项目投资13.6亿元，医疗服务一体化和分级诊疗全面施行，新农合参合率达99%。公共文化服务体系示范项目通过国家验收，文化先进县创建达标率全省第一，文化产业增加值年均增长10.8%。城镇居民人均住房31平方米、绿地13平方米。坚持安全发展，深化平安创建，“破小案暖民心”“三个精准防范”和交通安全联勤联动体系成为全国公安工作品牌，公众安全感连续五年保持全省前列。

  各位代表，过去六年工作的成绩与进步，是在市委的坚强领导下，全市各条战线共同努力的结果。我代表市人民政府，向全市广大干部群众、驻安部队指战员、武警官兵和政法干警，向大力支持政府工作的各位代表、各位委员和社会各界人士，致以崇高的敬意和衷心的感谢!

  六年来的奋斗历程启示我们：要把党的领导作为根本保证，坚决贯彻中央、省委和市委决策部署，营造心齐气顺、同心同向、干事创业的良好氛围；要把追赶超越作为目标定位，欠发达是我们的基本市情，但决不能成为安康永久的标签，必须对标一流，立愚公之志，创跨越之业，推动各项事业争先进位；要把改革创新作为动力之源，墨守陈规跟不上时代发展,封闭僵化实现不了追赶超越，必须发挥改革的突破性和先导性作用，强化科技创新的引领作用，为加快发展注入强劲动力；要把保护生态作为立市之本，环境就是民生，青山就是美丽，蓝天也是幸福，必须坚定绿色循环发展不动摇，着力打造绿水青山就是金山银山的实践典范；要把改善民生作为根本追求，突出脱贫攻坚，办好民生实事，促进社会公平正义，让全市人民生活更美好；要把责任担当作为成事之要，激发敢于担当的气魄，弘扬埋头苦干的精神，增强善抓落实的才干，迈出追赶超越的更大步伐。

  在肯定成绩的同时，我们也清醒认识到：思想观念滞后仍然是发展的最大障碍，一些地方和干部动力不足、顾虑过重、守成求稳，缺乏敢想敢干、创新突破、走在前列的勇气和魄力，工作过得去但过不了硬；经济下行压力还在持续，稳增长调结构面临较大困难；开放创新体制机制不活，产业支撑能力不强，科技、人才、资本等要素短缺，资源环境约束更紧，实现新旧动能转换难度大；社会保障和公共服务水平还有较大提升空间，脱贫攻坚和全面小康任务艰巨。面对这些困难和问题，我们决不回避，必须尽责担当，主动作为，切实解决。

  二、今后五年工作的总体要求和主要任务

  未来五年是加快建设西北生态经济强市、全面建成小康社会的关键阶段，推动经济社会持续健康发展意义重大、责任重大。总体要求是：深入学习贯彻习近平总书记系列重要讲话特别是来陕视察重要讲话精神，按照“五位一体”总体布局和“四个全面”战略布局，紧扣“追赶超越”定位和“五个扎实”要求，积极践行新发展理念，认真落实市第四次党代会部署，围绕实现“五个显著提升”和构建“七个新格局”，以提高发展质量和效益为中心，以深化供给侧结构性改革为主线，实施创新驱动、开放引领、生态强市、城乡一体、民生优先发展五大行动，推进新型工业化、新型城镇化、农业现代化、信息化融合发展，加快工业化初期向工业化中期、传统农业向特色现代农业、城乡二元向城乡一体跨越，加快建设西北生态经济强市，同步够格全面建成小康社会。

  预期目标是：生产总值年均增速高于全省2个百分点，固定资产投资和规模工业增加值年均增长15%，社会消费品零售总额年均增长12.5%，2019年现行标准下农村贫困人口全部脱贫、贫困县全部摘帽，2020年与全省全国同步够格进入全面小康社会，2021年生产总值超过1500亿元，经济综合实力和群众生活水平再上新台阶。

  （一）实施创新驱动，全面提升经济综合实力。把创新作为引领发展的第一动力，坚持以科技创新为核心，加强政策引导和专项投入，支持企业发挥主体作用，实施“产业、企业、平台创新”工程，在富硒产业等重点领域实施一批技术攻关项目，提高科技对经济发展的贡献率。以安康高新区为引领，协同“三区两园”“飞地经济园区”联动，构建“1+6”创新发展平台，打造秦巴区域自主创新高地和最具活力增长极。加快以“互联网+”为载体的产业融合，构建以生态友好型产业为主导的绿色循环产业体系，培育富硒食品、包装饮用水等产值过千亿元产业集群，建设国家富硒产业基地；实施全域旅游发展三年行动计划，构建“一心两廊三区”发展格局，打造全国知名的健康养生旅游目的地。持续推进大众创业、万众创新，积极发展混合所有制经济，坚定支持民营经济做大做强。切实加强质量标准体系和品牌建设，争创一批占据制高点的行业标准和国家标准，培育一批有竞争力的知名品牌，争创国家地理标志产品保护示范区。

  （二）加强统筹协调，构建现代新型城镇体系。遵循城市发展规律，优化城镇空间总体布局，强化“多规合一”，促进“产城融合”，构建城乡一体发展格局。强化中心城市辐射带动，实施“湖城一体、疏解江南、重心北移”战略，力促江北城区新增10万城市人口，建设区域中心城市，打造“秦巴明珠”。统筹城乡区域发展，加快月河川道城镇带、汉江生态经济带建设，促进汉滨、恒口、汉阴、石泉一体化发展，力促旬阳撤县设市、恒口设区。抓好9县城、28个区域中心集镇和新型社区、美丽乡村建设，打造2-3个高品质小城市，培育一批产业带动型特色小镇和生态田园式美丽乡村，构建现代新型城镇体系。坚持以人为本建设与严格依法管理相结合，深化城市管理体制改革和机制创新，加快建设智慧城市。

  （三）坚持绿色发展，建设生态宜居的美丽安康。建设美丽安康是我们的责任，也是打造西北生态经济强市的根本。要坚定不移走生态优先、绿色循环发展之路，争创国家生态文明试验区，确保生态环境质量持续良好，加快生态优势向发展优势转化。强力推进“增绿、护水、治污、防霾”，以对历史和子孙后代负责的担当，加大生态建设与环境保护力度；五年内全市森林覆盖率达到70%，完成城市污水处理厂提标改造，建成沿江沿河35个重点镇污水垃圾处理设施，实施400个农村环境综合整治项目，地质灾害得到有效治理，汉江水质稳定达标，城乡居民饮水安全，空气质量继续保持全省前列。加强森林资源、野生动植物保护，实施秦巴山地、汉江及其支流采石采沙和尾矿专项治理，依法打击乱建滥采行为，切实维护生态安全。严格执行环境准入制度，促进生产、流通、消费过程减量化，推进清洁生产技术创新和应用，提升绿色循环发展质量效益。建成国家健康城市、森林城市和省级文明城市、环保模范城市。

  （四）突出开放引领，打造陕西向南开放桥头堡。强化全球视野和战略思维，把安康置于全国和世界经济大格局中谋划，敢于和善于面向国内外两个市场，组织两种资源为我所用。发挥区位优势，在江汉、关天、成渝经济圈和城市群发展中找准位置、寻求机遇，搭乘“一带一路”、长江经济带发展和武汉、成都、重庆、西安等国家中心城市建设的“顺风车”，以更大的气魄和胆略为开放发展创造条件，形成全方位、高层次、多领域开放发展格局。围绕2017年西康高铁开工、2018年安康新机场首飞、2020年县县通高速和汉江主航道通航目标，强力推进一批重大交通项目，加快构建综合立体交通走廊；全面提升电力、通讯、网络宽带等基础设施承载能力，同步构建“一心两轴多点”、辐射川陕鄂渝的智能物流网络，建成秦巴区域交通枢纽和商贸物流中心，全面优化开放合作的基础条件，使安康成为西北地区通江达海的重要节点。

  （五）坚持共建共享，提升人民群众幸福指数。实施民生优先发展战略，为实现基本公共服务均等、社会公平正义与和谐稳定而用心用力。坚持以脱贫攻坚统揽全局，强化责任担当,激发内生动力，促进社会协同，凝聚攻坚合力；突出产业扶贫与移民搬迁，力促经营主体、资金支持、政策保障、结对帮扶全覆盖，2018年宁陕县、镇坪县、旬阳县脱贫摘帽，2020年实现农村贫困人口不愁吃、不愁穿和义务教育、基本医疗、住房安全有保障。坚持普惠性、保基本、均等化和可持续原则，统筹推进教育、卫生、文化、体育、社保等各项事业发展，2018年所有县区达到较高水平的国家义务教育发展均衡县标准，2021年完成现有棚户区和农村危房改造，文化、体育事业全面发展，覆盖全生命周期的“大健康”服务体系基本形成。加强和创新社会治理，全面建设美丽安康、平安安康、健康安康。

  三、2017年目标任务和重点工作

  全市经济社会发展主要预期目标为：生产总值增长10.5%，规模工业增加值增长15%，全社会固定资产投资增长16.5%，社会消费品零售总额增长13.5%，财政一般预算收入增长6%，新增城镇就业2万人，城乡居民收入分别增长9%和10%，万元GDP能耗下降3%，303个贫困村15.7万贫困人口实现脱贫。

  （一）扩大总需求，积聚发展新动能

  聚力扩大有效投资。实施“千亿投资”工程，抓实220个市级重点项目，完成年度投资350亿元。创新政府投融资方式，加快补齐基础设施、产业发展、生态环保、社会民生等领域的短板。加快推进平镇和安岚高速、国省干线提等、汉江综合整治、月河补水、洞河水库、瀛湖治理、750千伏输变电工程和农村电网改造、光网城市和光纤到村等重大基础设施项目建设，抓好汉阴凤凰山隧道等项目前期工作。实施127个产业项目，年度投资120亿元，重点抓好天贸物流城二期、钒氮合金二期、旬阳烟厂技改和旬阳、白河电站、装配式建筑等重大产业项目。统筹推进移民搬迁、棚户区改造、保障性住房和公共服务等民生项目。抢抓中省政策机遇，适应市场需求，切实加强项目前期工作，提升项目谋划水平。加强银企对接，拓展融资渠道，新开工PPP项目15个以上。

  加快发展新经济。围绕提高供给质量，快速跟进信息技术发展，积极运用互联网、大数据、云计算等现代科技，推动新技术、新业态、新模式、新产业快速成长。大力培育信息经济，实施“互联网+”行动计划，推进网络宽带、“多表合一”等信息基础设施建设。务实推进与上海复星、阿里巴巴、中兴、太极等企业合作，力促高新区电子信息、大数据产业园等项目建设，培育远程医疗教育、网上文化娱乐等数字服务新业态。鼓励发展分享经济，突出交通出行、生活服务、知识技能等领域，支持企业、个人依托互联网搭建共享平台，推动营销模式变革和闲置资源转化。发展壮大消费经济，加强城市商圈、商业综合体、各类专业市场及配套设施建设，支持天一城市广场、恒口500万吨货场、汉城国际商业街等项目加快推进。加快高新金融服务聚集区建设，积极创建绿色金融示范市，促进银行、证券、保险、基金、信托等业态发展。积极培育“文化+”新业态，新增规上文化企业15户，文化产业增加值增长15%。

  （二）聚焦融合发展，加快产业转型升级

  力促工业化与信息化融合发展。以信息技术为核心支撑，推进与工业生产的融合应用，增强工业整体竞争力。支持中科纳米、先进储能、帝奥电梯等企业加强研发，延长产业链条，提升终端产品品质。以推进智能制造为主攻方向，支持奥邦锻造、南水汽配、通用机械等企业转型升级，提升生产系统集成化和关键产品智能化水平，培育市场竞争新优势。适应个性化、多样化需求，开拓创新，主动出击，在培育3D打印、无人控制技术、高端智能装备、物联网基础设备等特色产业上求突破。下大力促进实体经济发展壮大，加强重点企业、困难企业帮扶，实施降低企业成本行动，打好减税降费、简政放权、银企合作组合拳，激发市场与资本活力。培育规上工业企业40户、产值过5亿元企业10户。

  做优做强生态富硒特色产业。围绕推进农业供给侧结构性改革，实施富硒产业发展行动计划，以茶叶、魔芋、包装饮用水、生猪等产业为重点，加强产品研发和市场开拓，整合资源、品牌、项目和人才，每个产业培育2-3户龙头企业、一批新型经营主体和专业技术人员，延伸产业链条，提升产品价值，增强核心竞争力，打造一批知名品牌。加快富硒食品产业园建设，依托富硒产品研发中心、国家地方联合工程实验室和富硒产品开发与质量控制重点实验室，务实推进与科研院校合作，加强关键技术研发，力促在富硒产品质量标准、生产加工技术等领域出成果、见成效。建立富硒产品可追溯和全过程质量安全体系，加快制定最严谨的富硒食品安全标准，积极创建出口食品农产品质量安全示范区。支持发展中医药产业。加快农村信息网络基础设施建设，实施“互联网+农业”计划，构建多业态、多功能的现代农业体系，促进农林旅游融合发展，多环节多层次增加产业效益。

  以全域旅游促进服务业增速提效。强化全域全景和融合发展理念，推动旅游与特色城镇、美丽乡村、现代农业、文化体育融合，完成4A景区整改提升和凤凰山景区等10个旅游项目策划规划，突出抓好中心城市“一江两岸”、瀛湖文化旅游产业基地、秦岭、巴山等景区景点提升建设，积极推动南宫山创建5A景区。加快推进汉江生态观光旅游带、月河川道现代农业休闲园和文化旅游特色小镇、乡村旅游等重点项目，支持争创全域旅游发展示范县，打造“十镇十村百户”示范典型。以全域旅游为促进，放大生态比较优势，加快发展大健康产业，构建以富硒食品、自然养生、健康养老、康养服务、体育运动等为支撑的健康产业体系和服务体系，建设亚高原训练中心和特色运动小镇，培育生态康养产业集群，打造区域健康产业发展示范区。大力发展电子商务、现代物流、中介服务等新兴服务业，改造提升商贸零售、住宿餐饮、交通运输等传统服务业。培育限上商贸企业45家，完成社会消费品零售总额284亿元。

  提高产业集群集约发展水平。坚持把园区作为要素和产业集聚的载体，加快基础设施和公共服务配套，深化体制机制和政策环境创新，打造最具活力的创新开放发展平台。安康高新区要对标一流的国家高新区，围绕建设创新创业示范区、高新产业聚集区和现代城市新区，进一步加大基础设施和服务配套建设力度，特别要加快教育、卫生、体育、文化等社会事业发展，促进循环产业、创新资源和人气聚集；实施智能终端电子信息、小巨人产业园等30个重点产业项目，加快金融服务聚集区、硒谷动力创业园等建设进度，提升7个国字号“众创空间”和创业孵化园功能，增强自主创新和引领创新能力，在经济增长、财税收入、进出口等方面提高对全市的贡献率。恒口试验区要围绕镇级市、建制区的目标定位，坚持多规合一、产城融合思路，突出抓好生态农业、文化旅游、商贸物流等重点产业发展，加强恒口古镇保护开发，推进阳安铁路复线、安康机场、月河综合整治、移民搬迁等重大项目建设，全面提升基础设施、市政配套和教育、卫生、文体等公共服务水平。瀛湖生态旅游区要紧扣“湖城一体”发挥龙头带动作用，深化开放合作，强化选商选资，加快旅游综合服务区、吉河绿色康养休闲区和金螺岛、玉兴岛、翠屏山庄等项目建设，推进水上竞技运动设施、帆船赛艇运动基地、沿湖马拉松和自行车赛道建设；围绕“梦幻瀛湖”策划夜间游乐项目，促进旅游、文化、体育深度融合，彰显湖光山色、梦幻水乡独特魅力；协同汉滨、岚皋、紫阳加强瀛湖湖泊治理，确保水质安全；严格规划管控，严惩乱批乱建。旬阳高新区要围绕撤县设市、工业转型和城市品质提升，着力创新体制机制，坚持高起点规划、高标准建设、高水平运营，培育壮大先进制造、新型材料、生物制品、包装饮用水等产业，全面提升新型工业化、新型城镇化水平，努力在率先发展中走前列、树标杆。县域工业集中区和“飞地经济园区”要进一步完善规划，统筹空间、规模、产业结构和生产、生活、生态布局，实施错位发展，在土地集约节约、标准化厂房建设和配套服务上下功夫，重点支持科技含量高、成长性强的企业落地；严格按投资强度和产出效益供应土地，防止圈占土地和低水平重复建设；宁陕、紫阳、岚皋、镇坪、白河5县要加强“飞地经济园区”规划管控和配套建设，在招商引资、项目落地、发展成效上比干劲、争一流。现代农业园区要以绿色、循环农业为发展方向，把扩大富硒农产品供给作为重点，深入推进“五个一”提升工程，加快与二、三产业融合，培育一批带动性强的龙头企业，壮大职业农民队伍；完善农业园区包联贫困户工作机制，每个省级园区至少包联120户、市级园区不少于60户、县级园区30户，带动农业增效和农民增收。支持县区争创省级经济技术开发区及各类创新示范区。

  （三）建设品质之城，释放新型城镇化潜力

  加快完善中心城市功能。深入推进“湖城一体、疏解江南、重心北移”，加快建设区域中心城市和生态宜居品质城市。围绕“湖城一体”，系统谋划瀛湖-中心城市-安康湖（汉江旬阳库区）一体化发展，融合山水、人文等元素，打造城在湖中、湖城一体的特色旅游观光区；规划建设“一江两岸环江百里”，统筹实施文化旅游、体育运动、亲水体验、休闲观光等重点工程，建设环江自行车和国际马拉松赛道；强化特色小镇理念，坚持规划引领，以基础配套、功能提升为切入点，推进张滩、县河、石梯片区发展，建设城东新区；实施安康大道、江北大道、瀛湖大道、环城南路改造和黄沟、七里沟、石张大道、西坝防洪工程建设，加快西城文化广场、西津公园、东坝内环路及排涝工程、汉江公园三期、黄洋河公园等项目建设。围绕“疏解江南”，实施“退、转、改、停、禁”，逐步把江南城区打造成商务宜居区。“退”，就是继续实施“退公还公”，用三年左右时间整合腾退一批市、区机关单位公共资产，全部用于绿地、停车场、健身休闲场所等公共服务设施。“转”，就是推进劳动密集型企业、综合批发市场、客货运站（场）和优质教育、卫生、文化、体育资源向江北和城东新区转移，实施市级机关事业单位和各类经济组织、社会组织北迁，推进中心城市第二水源地建设，启动江南污水处理厂搬迁扩建。“改”，就是围绕改善群众生活环境和提高城市品质，有序推进东坝、西坝、西关、兴安、高井、东关等片区棚改，完成2.6万户棚改任务；加快实施金州路、兴安路二期和巴山路、金州北路、中心广场和城区背街小巷提升改造。“停”，就是依法实施最严格的规划管控，停止江南建成区新的住宅开发项目土地交易和规划审批，停止江南城区除危房改造之外的私人建房审批，严控江南城区开发建设强度。“禁”，就是在城市规划区内禁止乱占土地和乱搭乱建，禁止秸秆焚烧和粘土砖生产，禁止燃放烟花爆竹和机动车鸣笛，禁止违法土葬和乱埋乱葬，适时启动江南主城区禁行摩托车。围绕“重心北移”，突出产城融合，狠抓基础配套，实施长春路、北环线、月河快速干线、机场干线等控制性工程和医养中心、工人俱乐部、体育运动场馆、青少年足球运动中心、新罗公园、鹭湾中央公园等重点项目；加快张岭、火车站等片区棚改；在机场、高铁站区域内规划建设客（货）运中心和专业市场；积极推进空港新城、月河口汉江大桥、游客综合服务中心、科技馆、美术馆、青少年活动中心等项目前期工作。

  深入推进城乡一体化。统筹月河川道城镇带、汉江生态经济带和县城、重点镇、新型社区以及美丽乡村建设，以“三区两园”和“飞地经济园区”为支撑，以中心城市为龙头，以石泉、汉阴县城和恒口试验区为节点，加快月河川道区域一体化发展。实施标准化县城创建，完善配套功能，提升综合承载能力。深入推进特色小（城）镇建设，坚持“一镇一特色”，加快16个全国重点镇、1个省级示范镇、3个省级文化旅游名镇（街区）、12个县域副中心镇、4个美丽乡村标准化建设，力促2个以上特色小（城）镇纳入国家支持计划。加强汉滨双柏、旬阳湛家湾、石泉长兴等传统古村落保护。以建设美丽宜居乡村为抓手，实施改善农村人居环境“九大工程”，抓好10个旅游扶贫示范村和10个标准化中心社区建设。

  以精细化管理提升城市品质。探索建立城镇“规划、建设、管理相统筹，权力、责任、投入相一致”的管理体制和运行机制。完成城管执法体制改革，坚持市区一体，推动重心下移，权责一致，高效联动。加强城市管理投入和执法队伍建设，依法治理道路交通、市场经营、市容环境方面的违法违规行为，创建安康大道、高新大道、滨江大道、金州路、兴安路、解放路、大桥路、育才路8条文明示范街区。坚持教育引导、示范引领、严格执法相结合，持续推进“城市文明提升”行动计划，让内在修养、公德自觉、善行义举融入市民生活、成为行为习惯，浸润城市的每一个角落。

  （四）统筹开放合作，增强改革发展内生动力

  深化对外交流与合作。实施开放发展行动计划，主动融入“海外陕西”发展，发挥1.3万安康籍海外华人华侨资源优势，加强与美国派克威尔、澳大利亚班优等国际友好城市合作，力促在欧洲、中亚地区再建立1至2个友好城市。积极筹建安康二类口岸和海关出入境检验检疫机构，支持企业拓展海外市场、吸引境外投资，推动安康特色产品走出国门。深化与毗邻城市、友好城市、对口支援地区和中省帮扶单位、科研院校交流合作，继续推进驻华和驻外使节安康行活动。牵头开展汉江流域水质保护、生态经济发展等区域性主题研讨和文化交流、体育赛事等活动，提升安康影响力。以更大力度推动安康人“走出去”，分批选派千名公职人员到党校（行政学院）、高校等机构和发达地区培训学习；以企业家、产业大户、职业农民、村组干部为重点，组织千名经营管理人才、专业技术人才和乡土人才走出去学习培训。探索建立教育、卫生、文化、体育等事业单位编制人事动态管理办法和重大贡献奖励激励机制，落实科技成果转移转化政策措施。统筹生态文明、社会治理等各领域改革创新，增强市场活力、社会活力与发展活力。

  精准实施选商选资和聚智引才。大力引进优质企业、优秀人才、先进技术和装备，助推产业转型升级。实施重大招商项目攻坚行动，积极促成央企、陕企和优秀民企走进安康，主动对接安康籍在外企业家回乡创业；抢抓军民融合发展的机遇，力促军工企业投资安康。积极跟进“互联网+”“健康中国”等国家政策，在电子信息、智能制造、文化旅游、现代物流、金融、新能源、大健康以及文化、体育等产业领域，深入研究、深度策划和推介一批优质项目，精准对接、务求实效。加强社会诚信和个人信用体系建设，进一步优化投资环境，全年招商引资到位资金500亿元。持续实施“百千万”人才工程，建立柔性引才机制，探索引进高端人才和专门人才的特殊措施，让更多优秀人才和创新团队来安创业。

  （五）依托资源优势，打造绿水青山生态典范

  突出保护为先，全面加强生态建设。持续推进“绿化安康”行动，加强天然林保护，提升退耕还林质量，完成造林绿化50万亩。实施山水林田湖保护和修复工程，严格控制矿产资源开发，严守耕地红线。突出沿江、沿河集镇社区和城乡结合部，扩大污水垃圾处理设施覆盖面，确保汉江水质稳定达标、城乡居民饮水安全。加大“气化安康”“电能替代”力度，加快推进改电改气，大力发展清洁能源。倡导绿色、低碳生活，让保护生态环境、建设美丽家园成为全民自觉行动。统筹绿色工厂、绿色产品、绿色园区和绿色供应链发展，发挥全省山林经济示范效应，加强生态产品有效供给，壮大绿色循环产业，培育生态经济新业态。

  坚持严字当头，强力推进综合治理。坚持源头严防、过程严控、后果严惩，严格执法监管和监测预警，突出抓好水、大气、土壤污染防治，对各类环境违法违规行为“零容忍”。围绕建设汉江绿色生态廊道，坚持和完善“河长制”，推动全民和全社会参与水污染防治；加强汉江综合治理、重点流域水质监测和地下水资源、自备水源管理，实施月河整治和丹治Ⅲ期工程。打好“减煤、控车、抑尘、治源、禁燃、增绿”组合拳，稳定保持空气质量全省领先。深入推进农村人居环境综合整治，全面推行农业测土配方施肥，开展工业能耗减量行动，实施公共机构节能计划，推进中心城区和区域范围内垃圾焚烧利用。强化环境保护“党政同责、一岗双责”，实行目标考核、责任追究和“一票否决”，坚决守住蓝天绿地，精心呵护一江清水，让绿水青山成为金山银山。

  （六）办好民生实事，不断增强人民群众获得感

  坚决打赢脱贫攻坚战。脱贫攻坚任务艰巨、使命光荣，也是重大发展机遇。要强化系统思维，既努力实现脱贫目标，更为农村长远发展打好基础。突出抓好产业扶贫和移民搬迁，力促每个贫困村有一项主导产业、一个专业合作组织，务实推进电商、旅游、光伏扶贫和资产收益扶贫试点；建立移民搬迁市县镇三级领导抓点、责任部门包联工作机制，加强集中安置点基础设施和产业配套，完成移民搬迁6.3万户17.8万人，让产业成为脱贫攻坚的关键支撑，让搬迁成为群众告别贫困的新起点。加大教育扶贫推进力度，确保每个贫困家庭的孩子都能接受良好的教育，坚决阻止贫困现象代际传递。整合各类涉农资金，加大金融扶贫投入，解决好农村交通、电力、水利等基础设施滞后和贫困群众安全住房等突出问题，加快光纤宽带进村入户，借助互联网激发农业、农村发展活力。

  全面提升公共服务水平。完善和落实就业创业优惠政策及配套措施，重点做好高校毕业生、移民搬迁户、城镇“零就业”家庭等群体就业帮扶工作。加强科技资源统筹，建立适应科技创新需求的人才管理和收入分配机制。坚持以促进义务教育均衡发展为重点，统筹推进学前教育发展、义务教育标准化、普通高中质量提升和职业教育基础能力建设，岚皋、紫阳通过国家义务教育均衡发展县验收；支持和规范民办教育发展。加强基层医疗卫生机构能力建设，提高公共卫生和计划生育服务水平；深入推进综合医改试点暨城市公立医院改革，完善医中有养、医养互助、签约服务等模式，加快构建全生命周期健康服务体系。加强公共文化阵地和人才队伍建设，抓好汉调二黄、紫阳民歌等非物质文化遗产保护利用，广泛开展群众文化活动与对外文化交流，推动建立南水北调中线城市文化联盟；坚持机关干部带头，力倡勤读书、读好书，促进全民阅读，建设书香安康。以筹备第十四届全运会为契机，完善城乡体育设施，积极发展体育产业，促进全民健身和竞技体育发展，办好第十七届汉江龙舟节。实施“全民参保”计划，推动社会保险全覆盖。

  深入推进“平安安康”建设。完善立体化社会治安防控体系，推行网格化管理，实施“雪亮工程”，坚决防范和打击各类违法犯罪，持续增强人民群众安全感。建立安全生产风险分级防控和隐患排查治理机制，严格实施道路交通、危化物品及其运输等重点领域安全监管，加强城镇消防安全设施建设，开展城镇老旧住房、农村危房排查治理，坚决遏制重特大事故发生。突出抓好防汛、防火和地质、气象灾害防范及应急处置，提升科学防灾减灾能力。落实“四个最严”要求，确保食品药品安全。依法规范信访秩序，完善联动协作机制，预防和化解社会矛盾。严格落实重大决策社会稳定风险评估制度，推进公共法律服务体系建设，全力防范经济、社会、自然风险，确保社会大局和谐稳定。

  各位代表，改善民生没有终点站，只有连续不断的新起点。我们要带着感情、责任和担当，持续办好人民群众最需要的实事，让城市更加文明，让社会更有温度，让幸福更具质感。今年重点抓好八大民生工程：

  一是创业就业工程：实施劳动力就业促进、大学生创业创新、能人返乡创业“三大行动计划”；在北京、西安等地开展安康籍大学生返乡创业对接活动，力促更多安康籍在外企业家和大学生回乡创业；办好中国创新挑战赛和“圆梦安康”创客大赛，建立“双创”带头人激励机制，提升众创空间和创业孵化平台功能，争创省级创业型城市；新增城镇就业2万人，免费技能培训2.5万人次，发放创业担保贷款4亿元。

  二是教育提升工程：围绕创建省级文明城市，加强公民道德建设，组织开展传统文化、文明礼仪、法律知识进基层、进社区、进校园等活动，提升城乡居民文明素质和社会文明程度。优化中心城区教育布局，加快安中高新分校、市一小高新分校、安康职业技术学院附属小学、高新初级中学、高新幼儿园建设，实施安康中学、汉滨高中、初中和枣阳小学等学校改扩建；推进市委党校二期建设；支持安康学院建特色学科、创一流学院；加快安康职业技术学院“一校两中心”建设，争创省级示范高职学院。新建和改扩建中小学、幼儿园235所，选派千名优秀教师参加国内外培训（境外培训50人左右）。

  三是卫生强基工程：坚持以人民健康为中心，全面提升公共卫生和医疗服务能力；推行县镇医疗服务一体化和责任医生团队“茨沟模式”，落实分级诊疗制度，推进医疗、医药、医保联动，提高社区医疗服务能力；新建和改造提升3个县级医疗卫生机构；加快安康职业技术学院附属医院、汉滨区卫生服务中心等项目建设，建成市公共卫生服务中心、市妇幼保健院和市中心医院、中医院全科医生培训基地，整合中医资源建设秦巴区域中医诊疗中心；加强卫生信息化建设，提升远程会诊系统覆盖面和服务水平；选派千名优秀医务工作者参加国内外培训（境外培训50人左右）。

  四是文体惠民工程：加快构建公共文化服务体系，借助信息技术创新服务模式，打造“文化大本营”服务品牌；实施文化人才“十强百优”计划，重点支持创作10部文艺精品；改造提升图书馆5个，新增图书5万册；加快汉江大剧院、香溪书院、移民博物馆建设，力促院地共建图书馆、西城文化广场建成投用；推进汉江公园、市体校提升改造；规划建设汉江全民健身长廊、市体育运动公园、银龙湾运动休闲中心、朝阳沟山地自行车赛道、瀛湖水上竞技运动设施及帆船赛艇基地和安康档案馆、美术馆；完善县级“三个一”体育设施，打造城镇社区15分钟健身圈。

  五是市政优化工程：启动中心城市“四网同线”改造，推进地下综合管廊建设，统筹实施供水排水、雨污分流、污水处理等项目；实施翠屏路、泸康大道、西津公园、北门广场、汉滨文化活动中心等项目建设；加大气化工程推进力度，力促中心城市和9县城气化率分别达到90%和60%；结合“一江两岸”、棚户区改造和城市干道建设，完善电力、通讯、供排水以及便民广播、治安警务、运动健身、绿化美化等配套设施；加快城区公共场所、大型商圈等重点区域免费WIFI全覆盖；拟将香溪洞风景区改为香溪公园并免费向市民开放。

  六是放心消费工程：聚焦民生领域和群众反映强烈的突出问题，开展消费市场秩序专项整治，让生产经营者不敢心存侥幸，让违法犯罪者付出高昂代价。组织开展放心消费创建活动，推出10-20个放心消费先进单位、示范街区、诚信企业和消费者满意企业（店铺）；在媒体设立“曝光台”，广泛发动群众监督，让假冒伪劣、以次充好、缺斤短两等消费欺诈行为无处藏身，让“诚信经营、放心消费”成为安康品牌。坚持把食品安全作为重大民生工程来抓，加快构建“从农田到餐桌”全过程全产业链追溯体系，全面治理“餐桌污染”，提高餐饮业质量安全水平，以严格的监管和教育引导，让原料生产者讲良心、加工经营者讲诚信，让绿色有机成为安康富硒食品、道地药材的标识，让纯正的安康味道成为放心消费的代名词。

  七是交通治理工程：针对中心城市拥堵问题，强化市区一体，突出重点区域和主要路段，加强综合执法管理；启动安康大桥北桥头、香溪路口、七里沟等交通组织建设；把公交优先发展作为长期战略，积极创建公交优先发展示范城市，建成江南公交总站和高新区长途汽车站，新投放公交车100辆，延伸加密公交班次，积极发展观光公交，推动公交向小区和背街小巷延伸，提高覆盖面和准点率，让公交成为市民出行的首选；倡导绿色、低碳出行，再投放公共自行车1000辆，让骑乘自行车成为一种时尚；全力推进停车场建设，新增停车位1000个；严格管控摩托车、电动车和各类代步车辆，坚决整治不守交规、非法营运、乱停乱放等行为。

  八是关爱扶助工程：推进15个社区老年人日间照料中心、100个农村幸福院和市残疾人康复中心建设，完善低保、五保、医疗等救助体系，分年度将全市2.49万户计划生育贫困家庭和“失独”家庭纳入特别救助范畴；整合助学项目和资金，保障进城务工人员子女入学，推动贫困家庭大学生资助全覆盖，决不让一个学龄儿童因贫困失学，决不让一个孩子因贫困上不了大学，确保未升学初高中毕业生一个不少地接受免费技能培训。支持红十字会、慈善协会、义工联等公益组织发展，建立志愿服务激励机制，壮大志愿者服务队伍，让爱心涌动秦巴汉水。

  四、务实推进政府自身建设

  重任千钧惟担当。新的历史使命对政府工作提出了更高要求，我们要把追赶超越的重担扛在肩上，把人民群众的忧乐放在心头，以法治为先、担当为要、廉洁为基，不断提高施政能力和服务水平，努力建设人民满意政府。

  全面加强依法行政。牢固树立现代法治意识，把政府工作全面纳入法治轨道，建立完善依法行政制度机制，深入开展“七五”普法，做学法、尊法、守法、用法模范，促进科学立法、严格执法、公正司法和全民守法，建设法治政府、法治安康。不断深化“放管服”改革，加快行政许可标准化，全面推行“双随机一公开”监管，建好用好市县政务中心、公共资源交易中心和投资项目并联审批系统，进一步精简会议文件，加快转变政府职能，全面提高行政效能。深化政府信息和政务公开，完善新闻发言人制度，加强政府网站、网上信访、市长信箱等平台建设，推进“安康发布”等客户端提质扩面；重视网民意见，善于在媒体监督下工作，善于运用媒体了解社情民意、发现矛盾问题、引导社会情绪、推动改革发展。

  强化责任担当落实。权力就是责任，责任就要担当，担当贵在实干。历史的使命、发展的重任和人民的期待，要求我们必须把尽责担当作为始终如一的根本。从政府班子自身做起，强化“责任在我、发展靠我”的担当，树立“功成不必在我、建功必须有我”的境界，拿出“踏石留印、抓铁有痕”的韧劲，凝聚全市人民的力量，变规划为行动，化蓝图为现实。政府各级领导干部要发挥示范引领作用，当好改革发展主攻手，勇做追赶超越带头人，挑得起重担子，啃得下“硬骨头”，做改革的促进派、发展的实干家。每个公职人员都要心往一处想、劲往一处使，困难面前不退缩，矛盾面前不回避，压力面前不低头，把作风、能力、水平体现在抓落实、促发展的实绩上。

  坚持为民务实清廉。深怀为民之心，牢记为民宗旨，做到依法用权、秉公为民、廉洁勤政。继续推行重点工作和民生实事公开承诺，说到做到，跟踪问效；办就办好，取信于民。深化行政效能建设，借助现代信息技术，推行订单式、精准式、并联式服务，用“明规矩”打破“潜规则”，让“办事不求人”成为常态。全面落实政府廉洁建设主体责任和“一岗双责”，坚持用制度管权管事管人，让权力在阳光下运行，主动接受人民群众监督，从源头上加强廉洁风险防控。坚持把纪律和规矩挺在前面，严格遵守党章党纪，持续用力转作风促政风带民风，树立为民务实清廉的政府形象。

  各位代表！人民赋予重托，奋斗创造未来。让我们在省委、省政府和市委的坚强领导下，坚定愚公移山的志向，砥砺勇于担当的品质，迈出追赶超越的更大步伐，为加快建设西北生态经济强市、全面建成小康社会而努力奋斗！
